# Human Dental Pulp Stem Cells Grown in Neurogenic Media Differentiate Into Endothelial Cells and Promote Neovasculogenesis in the Mouse Brain

**DOI:** 10.3389/fphys.2019.00347

**Published:** 2019-03-28

**Authors:** Jon Luzuriaga, Oier Pastor-Alonso, Juan Manuel Encinas, Fernando Unda, Gaskon Ibarretxe, Jose Ramon Pineda

**Affiliations:** ^1^Signaling Lab, Department of Cell Biology and Histology, Faculty of Medicine and Nursing, University of the Basque Country (UPV/EHU), Leioa, Spain; ^2^Laboratory of Neural Stem Cells and Neurogenesis, Achucarro Basque Center for Neuroscience, Leioa, Spain; ^3^Ikerbasque, The Basque Foundation for Science, Bilbao, Spain

**Keywords:** DPSCs, stereotaxia, endothelial, cell survival, nude mice, regenerative medicine

## Abstract

Dental pulp stem cells (DPSCs) have the capacity to give rise to cells with neuronal-like phenotypes, suggesting their use in brain cell therapies. In the present work, we wanted to address the phenotypic fate of adult genetically unmodified human DPSCs cultured in Neurocult^TM^ (Stem Cell Technologies), a cell culture medium without serum which can be alternatively supplemented for the expansion and/or differentiation of adult neural stem cells (NSCs). Our results show that non-genetically modified human adult DPSCs cultured with Neurocult NS-A proliferation supplement generated neurosphere-like dentospheres expressing the NSC markers Nestin and glial fibrillary acidic protein (GFAP), but also the vascular endothelial cell marker CD31. Remarkably, 1 month after intracranial graft into athymic nude mice, human CD31+/CD146+ and Nestin+ DPSC-derived cells were found tightly associated with both the endothelial and pericyte layers of brain vasculature, forming full blood vessels of human origin which showed an increased laminin staining. These results are the first demonstration that DPSC-derived cells contributed to the generation of neovasculature within brain tissue, and that Neurocult and other related serum-free cell culture media may constitute a fast and efficient way to obtain endothelial cells from human DPSCs.

## Introduction

The dental pulp is home to an active population of neural crest stem cells, which are responsible of renewing adult differentiated cells, such as odontoblastic cells and Schwann cells, within the pulp tissue (Kaukua et al., 2014; Aurrekoetxea et al., 2015). These cells are referred to as dental pulp stem cells (DPSCs) when extracted from permanent teeth, and as stem cells from human exfoliated deciduous teeth (SHEDs) when extracted from first generation teeth during childhood (Gronthos et al., 2000, 2002; Miura et al., 2003). Periodontal tissues also harbor different sources of stem cells with related characteristics (Marrelli et al., 2015; Chen and Liu, 2016; Avinash et al., 2017). All the aforementioned cell populations share stemness properties (Gronthos et al., 2000, 2002) and under the appropriate conditions are able to differentiate into several cell types, preferentially to those of mesenchymal lineages, like osteoblasts, chondroblasts, and adipocytes (Gronthos et al., 2002; Laino et al., 2005). Differentiation of SHEDs and DPSCs toward neural lineages has also been extensively reported in the literature in the presence (Zhang J. et al., 2016) or absence of scaffolds (Kim et al., 2012; Chang et al., 2014; Majumdar et al., 2016), especially when cells were cultured with DMEM/F-12 or Neurobasal medium supplemented with B27 and EGF/FGF growth factors (Widera et al., 2007; Arthur et al., 2008; Bonnamain et al., 2013; Gervois et al., 2015). However, a large debate remains as to whether those cells are genuinely neuronal or merely neuronal marker-expressing cells (neuron-like cells), or whether they can effectively integrate into the host brain neuronal connectivity after their graft *in vivo*.

Interestingly, DPSCs also have the ability to co-differentiate synergistically to osteoblast and endothelial cell phenotypes when cultured with fetal bovine serum (FBS)-containing media (d’Aquino et al., 2007; Hilkens et al., 2017). Despite the generation of a highly vascularized bone-like tissue after their subcutaneous graft *in vivo* (d’Aquino et al., 2007; Hilkens et al., 2017), the possibility to use of DPSCs as a source of young neovasculature has not yet been seriously considered with a view to the clinic. One of the underlying reasons is the need of fetal serum (10–20% FBS + α-MEM) which makes part of most endothelial differentiation recipes (d’Aquino et al., 2007; Arthur et al., 2008; Bueno et al., 2013). Although fetal serum is beneficial for cell survival and rapid cell expansion, its presence also favors the differentiation of DPSCs toward osteoblastic/odontoblastic lineages (Yu et al., 2010; Pisciotta et al., 2012). Moreover, cellular uptake of serum during *in vitro* culture might also cause host allergies and immune reactions against the transplanted cells (Gregory et al., 2006).

In this study, we explored the possibility to obtain endothelial cells ready to use for *in vivo* grafts starting from genetically unmodified human DPSCs. For this purpose, cells were extracted and cultured directly with a completely serum-free medium. We chose a commercial neural stem cell (NSC) growth medium with the presence of heparin, EGF/FGF growth factors, and B27 without vitamin A. This proliferation-supplemented culture medium is routinely used to expand neural stem and brain cancer stem-like cells. We found that this medium was also supportive for DPSC expansion, and importantly, it was also permissive for the generation of endothelial cells (mesoderm), without any need of scaffolds or the presence of serum. Furthermore, the addition of the differentiation supplement kit to this culture medium made DPSCs receptive to *in vitro* differentiation toward neuronal and astroglial (neuroectoderm) fates. When DPSCs were grafted into the brain of immunocompromised nude mice, they were able to integrate into murine vasculature differentiating toward endothelial and pericyte lineages without osteoblast/cartilage production. The clinical relevance is major, because (i) we generated a large endothelial cell population out of free floating DPSC dentospheres and (ii) we did not use fetal serum, which is known to be highly allergenic and responsible for the rejection of cell transplants in humans (Gregory et al., 2006).

## Materials and Methods

### Cell Culture and Cell Proliferation

Human third molars were obtained from healthy donors of between 19 and 45 years of age. Tooth samples were obtained by donation after written informed consent in compliance with the 14/2007 Spanish directive for Biomedical research, and the protocol was approved by the CEISH committee of UPV/EHU. DPSC isolation and culture were carried out as previously reported (Gronthos et al., 2000). Briefly, DPSCs were isolated by mechanical fracture and enzymatic digestion of the pulp tissue for 1 h at 37°C with 3 μg/mL collagenase (17018-029, Thermo Fisher Scientific, Waltham, MA, United States), and 4 mg/mL dispase (17105-041, Thermo Fisher Scientific, Waltham, MA, United States). After centrifugation at 1500 rpm for 5 min, cells were resuspended and underwent mechanical dissociation by 18-G needles (304622,BD Microlance 3). Then DPSCs were cultured in parallel with different types of culture media: (i): DMEM (Lonza 12-733, Basel, Switzerland) supplemented with 10% of inactivated FBS (SV30160.03, Hyclone, GE Healthcare Life Sciences, Logan, UT, United States), 2 mM L-glutamine (G7513, Sigma, St. Louis, MO, United States), and 100 U/mL penicillin + 150 μg/mL streptomycin antibiotics (15140-122, Gibco). (ii): Human Neurocult medium composed of Human Neurocult NS-A basal medium (cat# 05750, Stem Cell Technologies, Vancouver, BC, Canada) with Neurocult proliferation supplement (cat# 05753, Stem Cell Technologies, Vancouver, BC, Canada) or Neurocult differentiation supplement (cat# 05752, Stem Cell Technologies, Vancouver, BC, Canada) both at 9:1 ratio, and supplemented with Heparin solution 2 μg/mL (cat# 07980, Stem Cell Technologies, Vancouver, BC, Canada), EGF 20 ng/mL, and FGFb 10 ng/mL (Peprotech, London, United Kingdom) as previously described (Pineda et al., 2013) in the presence of antibiotics penicillin 100 U/mL and streptomycin 150 μg/mL (15140-122, Gibco). For NSC cultures isolated from Nestin-GFP mice, dissected hippocampi were removed with ice-cooled PBS-sucrose and processed as SVZ as previously described (Pineda et al., 2013). Cells were maintained at standard conditions in a humidified 37°C incubator containing 5% CO_2_. Neurosphere cultures were then passaged every 7 days by enzymatic disaggregation with Accutase (Sigma, St. Louis, MO, United States). We cultured DPSCs cells for 1 month and a maximum of four total passages in order to avoid cell aging issues.

The population doubling (PD) rate was determined by initial cell culture. Cells, neurospheres, and dentospheres were disaggregated, counted, and passaged at day 7. At each passage, cells were re-plated at the initial density and cultures were performed until passage 4. The PD rate was calculated as previously described (Pisciotta et al., 2012). All cells were seeded at the density of 6 × 10^3^ cells/cm^2^ and cultured for 1 week. Cell counting was performed after cell detachment or dissociation using an automated TC20 from Bio-Rad cell counter. The total number of cells estimation was calculated on three experimental samples for each type of culture.

For phospho-STAT3 inhibition experiment on Neurocult proliferation cultures, 25,000 cells were allowed to attach and growth as monolayer using a coating of laminin matrix (1:100, Sigma) during 3 days. Then, Stattic an irreversible Stat3 inhibitor (S7947, Sigma, St. Louis, MO, United States) was added to the medium at 0, 1, and 2.5 μM concentration for 72 h as previously described (Li et al., 2018) and then cells were fixed for immunofluorescence analysis.

### Flow Cytometry

Half-million DPSCs grown in either DMEM 10% FBS or Neurocult proliferation media were detached, disaggregated, and then incubated with PBS 0.15% BSA solution with 0.5 μg of CD31-FITC or IgG2a κ Isotype control (#303103 and #400107, respectively, BioLegend CS, United States) for 1 h at 4°C. After a wash with PBS 0.15% BSA cells were resuspended in 300 μL of PBS 0.15% BSA and analyzed using a FACS Beckman Coulter Gallios (Beckman Coulter Life Sciences, Indianapolis, IN, United States). The data were analyzed using Flowing Software 2.5 (University of Turku, Finland).

### Animals and Cell Graft

Consanguine c57bl6 litters from Nestin GFP mice and Athymic Swiss^nu/nu^ were used as hosts for murine and human *in vivo* graft purposes. Nestin-GFP neurospheres or DPSCs dentospheres in the active growth phase were disaggregated, washed, and collected in Neurocult serum-free media. Two microliters containing 100,000 cells were injected (0.5 μL/min) unilaterally into the dentate gyrus of the hippocampus at the following coordinates (from bregma): AP = -1.9, L = -1.2, and DV = -2 and -2.1. The cell transplantations were performed using a small animal stereotaxic apparatus (Kopf model 900) with a 10 μL Hamilton syringe and a 33-G needle (Hamilton, Bonaduz, Switzerland). Pre-operatory and post-operatory animal care were carried out as previously described (Jeitany et al., 2015). Animals were provided with food and water *ad libitum* and housed in a colony isolator maintained at a constant temperature of 19–22°C and humidity (40–50%) on a 12:12 h light/dark cycle.

### Immunostaining of Brain Sections and Cell Culture

Animals were deeply anesthetized with Avertin 2.5% and perfused with a 4% paraformaldehyde solution in 0.1 M sodium phosphate, pH 7.2, and processed as previously described (Pineda et al., 2013). In order to detect grafted genetically unmodified human DPSCs on mice brain, specific antibodies targeted to human Nestin (MAB1259, 1:200 R&D systems) (Jeitany et al., 2015), and human CD31 (BBA7, 1:200 R&D systems) were used. Pericyte staining was performed using of anti-CD146 (ab75769, 1:150, Abcam). Immunostaining of brain vasculature was made using CD31 (550247, 1:300 BD Pharmingen), laminin (L9393, 1:200 Sigma, St. Louis, MO, United States), and VEGF (ABS82-AF647, 1:200 Sigma, St. Louis, MO, United States) antibodies. Both NSCs and DPSCs, either in the form of dissociated cells or small spheres, were seeded into laminin-treated coverslips (L2020, Sigma, St. Louis, MO, United States) as previously described (Silvestre et al., 2011). After 3 days or 1 week, they were fixed by incubation with 4% PFA for 10 min at room temperature and permeabilized by incubation in 0.1% Triton X-100. They were then incubated overnight at 4°C with primary antibodies at the following dilutions: glial fibrillary acidic protein (GFAP) (MAB3402, 1:400; Millipore, Lake Placid, NY, United States), Nestin (NES, 1:200 Aves Labs), S100ß (Z0311, 1:1000, Dako, Glostrup, Denmark), NeuN (EPR12763, 1:200; Abcam, Cambridge, United Kingdom), doublecortin (DCX) (sc-8066, 1:200; Santa Cruz, Dallas, TX, United States), CD31 (550247, 1:300 BD Pharmingen, San Jose, CA, United States), anti-VEGFR (#9698S 1:800, Cell Signaling Technologies, Danvers, MA, United States) anti-STAT3 (phospho Y705) antibody (ab76315, 1:500 Abcam), anti-human-CD31 (F8402, 1:200, Sigma St. Louis, MO, United States). For both tissue sections and cell culture, secondary antibodies conjugated to Alexa 488, 568, and 647 Donkey anti-mouse, anti-rabbit, or anti-goat were incubated for 2 h and 30 min, at room temperature. Preparations were counterstained with DAPI and images were captured using a Leica SP8 confocal microscope at 40X magnification.

### Conventional PCR and Quantitative Real-Time PCR (QPCR)

RNA extraction from cell pellets, reverse transcription, and QPCR were performed as previously described (Uribe-Etxebarria et al., 2017). The molecular weights of the amplification products were checked by electrophoresis in a 2% agarose gel. All reactions were performed in triplicate and the relative expression of each gene was calculated using the standard 2^-ΔΔCt^ method (Livak and Schmittgen, 2001). *GAPDH* and *β-ACTIN* were used as housekeeper genes. Primer pairs used were obtained through the Primer-Blast method (Primer Bank) and they are listed in Supplementary Table S1.

### Western Blot

Dental pulp stem cells grown using either Neurocult proliferation medium or DMEM + 10% FBS and liver sinusoidal endothelial cells (LSECs) were counted, pelleted, and resuspended in a ratio of 20,000 cells/μL with RIPA lysis buffer (R0278, Sigma, St. Louis, MO, United States) supplemented with protease (11873580001; Roche) and phosphatase inhibitors (78420; Thermo Scientific) in order to obtain the same cellular concentration for the different type of cells and culture media. From this, 30 μg of protein were diluted in a mix of RIPA buffer and LDS Sample Buffer (NP0007; Invitrogen by Life technologies). Electroblot was performed as previously described (Jeitany et al., 2015). Phosphorylated-ERK and total ERK antibodies (both 1:1000, #4370 and #4695, respectively, Cell Signaling Technologies) and phospho-Stat3 and Stat3 (both 1:1000, #9145 and #9132, respectively, Cell Signaling Technologies, Danvers, MA, United States) were used to detect the angiogenic signaling pathways and Ponceau staining (P7170-1L, Sigma St. Louis, MO, United States) was used as loading control to detect protein in the charged lanes.

### Statistical Analysis

Comparisons between multiple groups were made using Kruskal-Wallis followed by Dunn’s *post hoc* test. Comparisons between only two groups were made using U-Mann–Whitney test or Student’s *t*-test. *p* < 0.05 was considered as statistically significant. Results were presented as mean ± SD or SEM. The number of independent experiments is shown in the respective section.

### Study Approval

The animal experiments were performed in compliance with the European Communities Council Directive of November 24, 1986 (86/609/EEC) and were approved by the competent authority (Diputación Foral de Bizkaia).

## Results

### Comparative Characterization of DPSC Growth and Neural Stem Marker Expression Using Neurocult NS-A Proliferation Medium

Previous research had shown that the dental pulp tissue of adult teeth contained neural-crest derived DPSCs, which expressed NSC markers such as Nestin and GFAP (Gronthos et al., 2000; Chang et al., 2014). DPSCs were fully viable after 1 month post-graft and capable of *in vitro* differentiating into mesenchymal cell lineages and generating a dentin pulp-like tissue complex, after *in vivo* subcutaneous transplantation (Gronthos et al., 2002). Our first aim was to evaluate whether Neurocult^TM^, a serum-devoid cell culture medium widely used to grow adult NSCs and progenitors, would be also permissive for the growth of DPSCs. For this purpose, we compared DPSC cultures grown with the standard medium DMEM/FBS and Neurocult proliferation medium. Interestingly, DPSCs cultured with Neurocult maintained the expression of the brain-derived neurotrophic factor (*BDNF*, Supplementary Figure S1), a neurotrophin involved in neurogenesis and neuron survival (Canals et al., 2004; Rossi et al., 2006). As Neurocult proliferation medium is specially designed to generate neurospheres from dissected and isolated brain neurogenic niches, we used control cultures of NSCs from mouse hippocampi, by adapting the protocol previously described (Pineda et al., 2013).

**FIGURE 1:**
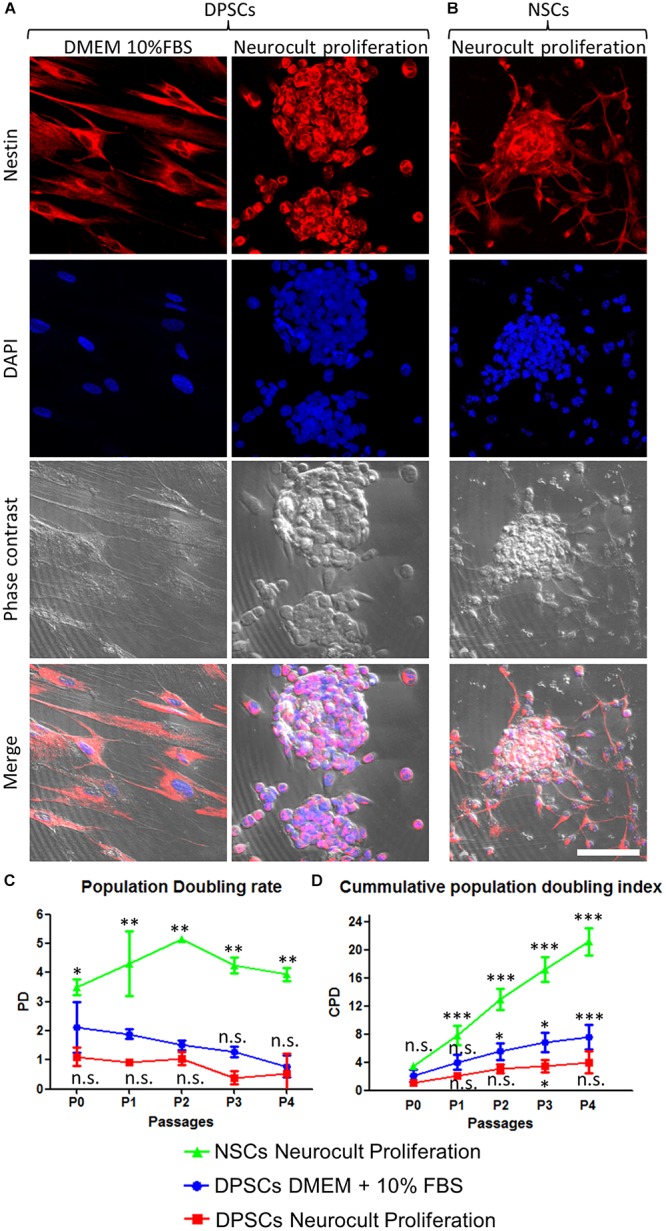
Human DPSCs grown in Neurocult proliferation culture media generate dentospheres similar to neurospheres from murine neural stem cells (NSCs). **(A)** Human DPSCs seeded in standard culture DMEM + 10% FBS acquire a flat morphology and adhere to the flask surface. However, when cultured with Neurocult proliferation media, DPSCs start to grow forming free-floating dentospheres. **(B)** Control neurospheres of murine NSCs. All cells are positive for the neural stem/progenitor marker Nestin. **(C)** Population doubling rates for human DPSCs grown either with DMEM + 10% FBS or Neurocult proliferation compared with murine NSCs. **(D)** Cumulative population doubling (CPD) of DPSCs seeded in standard culture DMEM+10%FBS or Neurocult proliferation media and NSCs cultured in Neurocult proliferation media. All different cell culture conditions were assessed in parallel (*n* = 3 for each) for a total of four passages and non-parametric Kruskal–Wallis with *post hoc* test was used (mean ± SD of two independent experiments ^∗^*p* < 0.05, ^∗∗^*p* < 0.01, and ^∗∗∗^*p* < 0.001). Scale bar 75 μm.

Both DPSC-derived dentospheres and NSC-derived neurospheres were grown in human and mouse-specific Neurocult^TM^ proliferation supplemented media, respectively. Once disaggregated from spheres, both NSCs and DPSCs were induced to attach to the platting surface using laminin-coated coverslips for 45 min, and they were immunostained against the intermediate filament Nestin, a marker expressed by NSCs both *in vitro* and *in vivo* (Reynolds and Weiss, 1992; Morshead et al., 1994). Both DPSCs grown with DMEM + 10% FBS or Neurocult proliferation media cells were positive for Nestin, as well as NSCs (Figure 1A,B).

We evaluated the growth kinetics of DPSCs cultured either using DMEM + 10% FBS or human specific Neurocult NS-A proliferation medium. NSCs were grown with mouse specific Neurocult proliferation medium. Human DPSCs showed a lower proliferative index than murine NSCs (Figure 1C,D). The PD rate in DPSCs showed no statistically significant differences between Neurocult proliferation with respect to DMEM + 10% FBS, but it showed a faster growth of NSCs (*p* = 0.0556 and *p* = 0.0037, respectively; one-tailed Kruskal–Wallis test; Figure 1C). However, the analysis of cumulative population doubling (CPD) showed that both DMEM/FBS and Neurocult DPSCs cultures had a parallel trend with steadily rising growth kinetics after consecutive passages without reaching significant differences until P3, where differences began to emerge between both conditions (*p* = 0.0273, one-tailed Kruskal–Wallis test; Figure 1D). It is noteworthy that DPSCs cultured using DMEM + 10% FBS grew attached to the flask plastic surface, even without any coating. Meanwhile, DPSCs in Neurocult proliferation had a progressively slower growth, and generated free-floating dentospheres during all the successive passages, as normally NSCs do.

**FIGURE 2:**
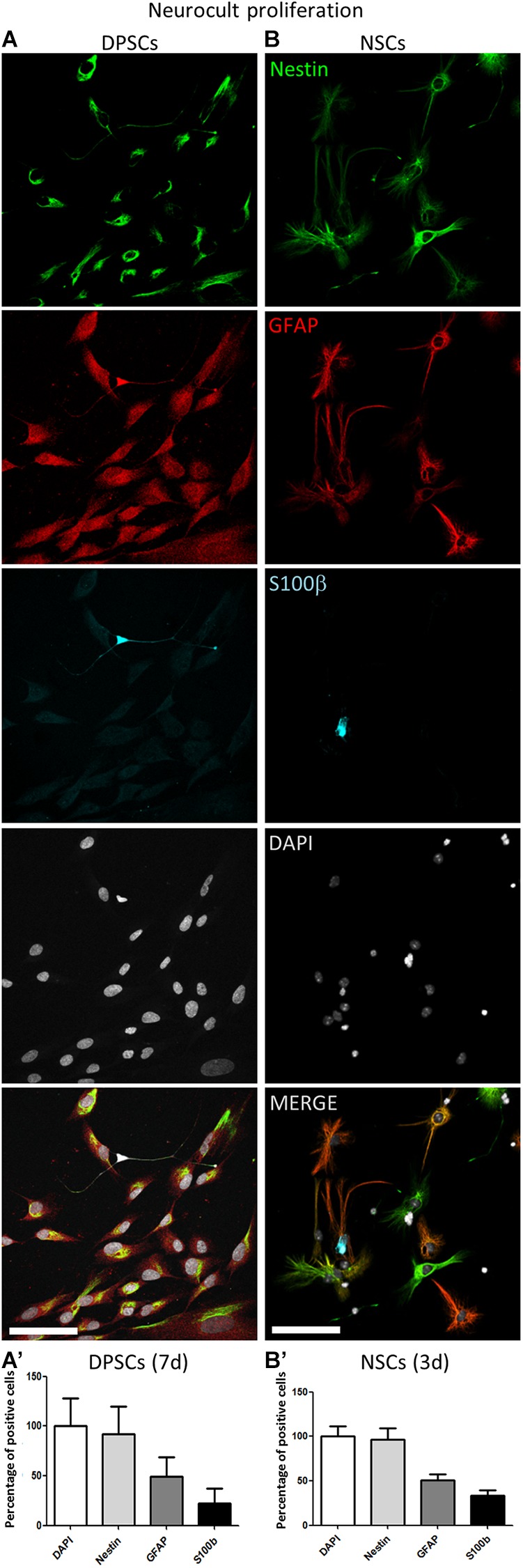
Human DPSCs grown in Neurocult proliferation share neural stem/progenitor markers with murine NSCs. **(A,A’)** Human DPSCs were allowed to grow for 7 days in laminin-coated wells in the presence of Neurocult proliferation media are Nestin positive and also express astroglial markers GFAP and S100ß (*n* = 230). **(B,B’)** Murine NSCs grown for 3 days (to avoid confluency) in the same culture conditions also express these astroglial markers in similar proportion (*n* = 242). Mean ± SEM of three independent experiments. Scale bar 75 μm.

Triple immunofluorescence analysis was performed against GFAP, Nestin, and S100β at 3 days post seeding for high proliferating NSCs, and at 7 days for the more slowly growing human DPSCs in Neurocult proliferation medium. Both cultures showed comparable proportions of label-positive cells in DPSCs and NSCs, for each of the three tested markers (Figure 2). Nestin-GFAP coexpression is a long-known and widely reported marker profile characteristic of NSCs (Davidson, 1994; Pineda et al., 2013), which was also found in DPSCs cultures using neuronal inductive media (Chang et al., 2014). The mature astrocytic marker S100β, whose expression in GFAP-expressing cells coincides with the loss of their NSCs potential (Raponi et al., 2007), could also be detected in a minority of cells, both in DPSC and NSC cultures. We determined that all human DPSCs cells were Nestin positive, in agreement with the results published by Chang et al. (2014) with a proportion of 69 ± 34% GFAP and 24 ± 17% S100β positive cells (Figure 2A,A’); 96 ± 18% murine NSCs grown with Neurocult proliferation media were Nestin-positive with a proportion of 52 ± 12% GFAP and 35 ± 10% S100β positive cells (Figure 2B,B’). Thus, DPSCs and NSCs grown in Neurocult proliferation media contained similar proportions of cells expressing NSC markers, and putatively astrocyte-committed S100β positive cells.

### DPSCs Grown Using Neurocult NS-A Proliferation Medium Increased the Expression of the Endothelial Marker CD31

**FIGURE 3:**
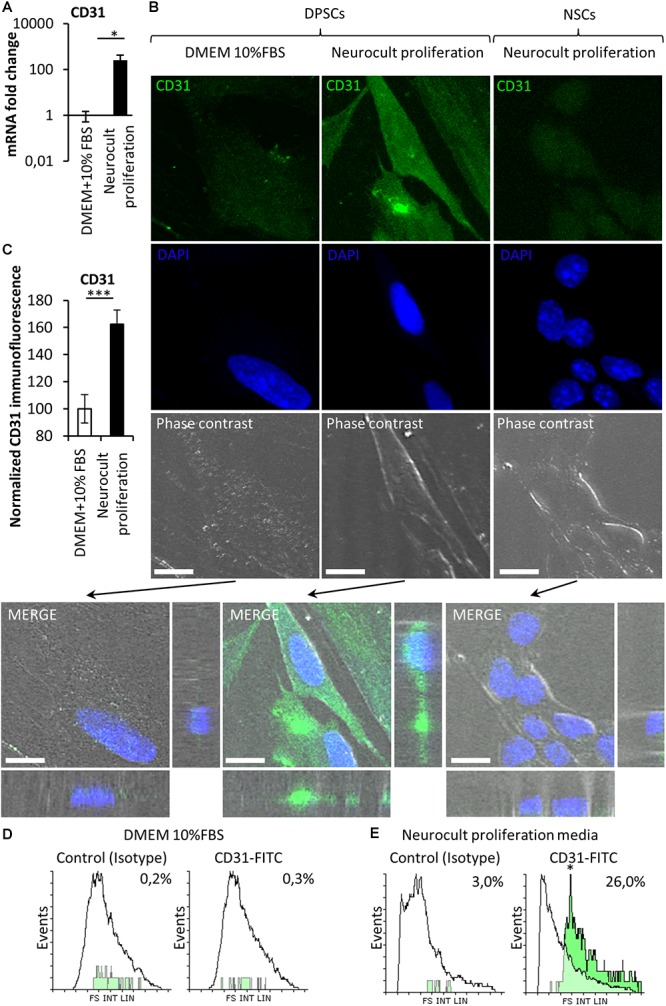
The endothelial marker CD31 is absent from NSCs whereas it can be enriched depending on the type of culture medium in DPSCs. **(A)** mRNA fold change of the endothelial marker CD31 on DPSC cultures depending on the culture medium: DMEM/FBS vs. Neurocult proliferation. Axis represented as logarithmic scale of CD31 expression from DPSC cultures of four different patients (mean ± SEM ^∗^*p* = 0.05, one-tailed Mann–Whitney test). **(B)** CD31 immunofluorescence and orthogonal projection of DPSCs cultured with (*left column*) DMEM + 10% FBS or (*middle column)* Neurocult proliferation media NSC (*right column*) cultured with Neurocult proliferation. Scale bars 10 μm. **(C)** Quantification of the immunolabeling signal intensity for CD31 (mean ± SEM of two independent experiments, *n* = 10 for DMEM 10% FBS and *n* = 10 for Neurocult proliferation, ^∗∗∗^*p* = 0.0008, one-tailed Mann–Whitney test), and **(D)** flow cytometry analysis for CD31 expression in DPSC culture with DMEM+10%FBS or **(E)** Neurocult proliferation media (*n* = 10.000 events for each condition; ^∗^*p* = 0.05, one-tailed Mann–Whitney test).

Stem cells from human exfoliated deciduous teeth and DPSCs grown in the presence of serum are able to differentiate into endothelial vasculature (d’Aquino et al., 2007; Sakai et al., 2010; Zhang D. et al., 2016), but it remained unknown whether this capability was dependent or not on the presence of fetal serum. Given that DPSCs grown in serum-free Neurocult proliferation expressed the stem cell marker Nestin, which has also been reported to be a marker of proliferative endothelial cell progenitors (Suzuki et al., 2010), we wondered if serum-free Neurocult proliferaton would also be permissive for the generation of endothelial cells by detecting the expression of *CD31* and *VEGF*, which are both markers of endothelial cells (Wong and Dorovini-Zis, 1996; Maharaj et al., 2006). Q-PCR of *CD31* mRNA expression in DPSCs cultured with Neurocult proliferation showed an increase of up to several orders of magnitude (249 ± 172 fold-increase of expression) with respect to cultures with DMEM 10% FBS (*p* = 0.0286; Mann Whitney test, Figure 3A). To confirm this result, in a next step, we decided to perform an immunofluorescence analysis for the endothelial marker CD31 followed by a confocal orthogonal cell reconstruction on laminin-coated slides, in both DPSCs grown in DMEM + 10% FBS and Neurocult proliferation conditions. Interestingly, murine NSCs derived from neurogenic niches were completely negative for CD31 staining (Figure 3B, right panel) and did not integrate into murine vasculature after intracerebral graft in consanguine mice, although they showed some projecting contacts to the brain vasculature, as previously described (Pineda et al., 2013; Supplementary Figure S2). By quantifying the fluorescence labeling intensity in images, we determined that DPSCs grown in Neurocult proliferation increased CD31 expression by 62 ± 10%, with respect to DMEM+10% FBS (*p* = 0.0008, one-tailed Mann–Whitney test, Figure 3C). It is known that laminin could induce a morphologic differentiation of DPSC to endothelial cells (Lawley and Kubota, 1989). In order to exclude this possibility, we cultured DPSCs without laminin as floating dentospheres and thereafter we run a flow cytometry analysis by labeling the cells either with CD31-FITC or the IgG control isotype. We detected an increase of up to 26% of CD31-FITC positive cell population in DPSCs when these cells were grown with Neurocult proliferation (Figure 3E). In contrast, the flow cytometry analysis only gave a residual value of 0.3% of CD31-positive DPSCs, when these cells were grown in DMEM + 10% FBS (Figure 3D). In conclusion, our results demonstrated that serum-free Neurocult proliferation medium promoted the expression of the CD31 endothelial-cell marker in DPSC cultures.

### DPSCs Grown in Neurocult NS-A Proliferation Medium Expressed the VEGFR2 Receptor and Showed an Increased ERK Pathway Activity

**FIGURE 4:**
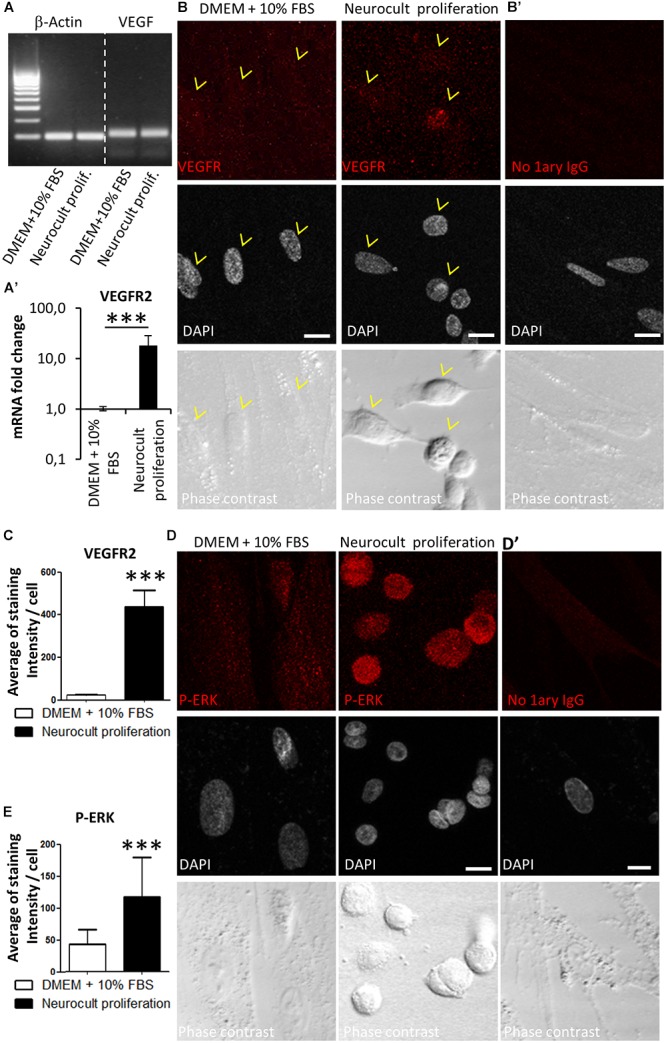
DPSCs grown in Neurocult proliferation increase the expression of VEGFR2 and activate downstream ERK signaling. **(A)** RT-PCR detection for VEGF, confirming its expression in both DMEM/FBS- and Neurocult-grown DPSCs. **(A’)** mRNA fold change of the endothelial marker *VEGFR2* on DPSC cultures depending upon the type of media: DMEM/FBS vs. Neurocult proliferation. Axis represented as logarithmic scale (mean ± SEM of three independent samples, ^∗∗∗^*p* = 0.0002, one-tailed, Mann–Whitney test). **(B)** Immunofluorescence of VEGFR2 receptor staining for cells grown during 7 days with DMEM + 10% FBS (*left*) or Neurocult proliferation (*right*). **(B’)** Control with no 1^ary^ antibodies. Scale bar 10 μm. **(C)** Quantification of distribution of the average amount of bright pixels per cell corresponding to VEGFR2 staining (*n* = 33 cells for DMEM + 10% FBS and *n* = 69 cells for Neurocult proliferation, ^∗∗∗^*p* = 0.0001, one-tailed, Mann–Whitney test). **(D)** Immunofluorescence of phospho-ERK1/2 showed an increase of staining for cells grown with Neurocult proliferation medium, in contrast to DMEM + 10% FBS DPSCs. Scale bar 10 μm. **(D’)** Control with no 1^ary^ antibodies. Scale bar 10 μm. **(E)** Quantification of distribution of the average of bright pixels per cell corresponding to phospho-ERK1/2 staining (*n* = 30 cells for DMEM + 10% FBS and *n* = 47 cells for Neurocult proliferation of three independent samples. ^∗∗∗^*p* = 0.0001, one-tailed, Mann–Whitney test).

It was previously reported that ERK signaling and VEGFR expression were required for endothelial differentiation of SHEDs (Bento et al., 2013). Because DPSCs grown with Neurocult proliferation expressed endothelial markers VEGF and CD31, we checked by RT-PCR that DPSC cultures were able to produce VEGF (Figure 4A). Next, we decided to test the presence of *VEGFR2* at mRNA and protein level by Q-PCR and immunofluorescence. DPSCs cultured with Neurocult proliferation showed 17 ± 10 fold-increase of *VEGFR2* mRNA with respect to cultures with DMEM 10% FBS (*p* = 0.0002; one-tailed Mann–Whitney test, Figure 4A’) and a corresponding increase of VEGFR2 immunostaining, which presented a characteristic punctuated pattern. We quantified immunolabeling intensity as the average of brighter pixels per cell above the autofluorescence level (signal without primary antibody). Neurocult proliferation DPSCs showed an average of signal of 416 ± 79 bright pixels per cell with respect to the average of 22 ± 3 bright pixels corresponding to cultures with DMEM 10% FBS (*p* = 0.001, one-tailed Mann–Whitney test, Figure 4B,C). Previous reports associated phosphorylation of ERK1/2 protein to endothelial cell differentiation of SHED (Bento et al., 2013), in addition to cell proliferation in a large variety of cell types (Cobb, 1999). Interestingly, DPSCs cultured with Neurocult proliferation clearly showed an increased pERK staining (Figure 4D, middle) with respect to DPSCs cultured with DMEM 10% FBS (Figure 4D, left). No staining was found in controls without primary antibody and a pixel signal threshold was set to determine and subtract background (Figure 4B’,D’). Quantification of pERK immunolabeling was made by averaging bright pixels per cell above the autofluorescence level (signal without primary antibody). Neurocult proliferation-grown DPSCs showed an average of signal of 116 ± 61 bright pixels per cell with respect to the average of 42 ± 23 bright pixels corresponding to cultures with DMEM 10% FBS (*p* = 0.001, one-tailed Mann–Whitney test, Figure 4D,E).

### DPSCs Grown in Neurocult NS-A Proliferation Presented a Concurrent Activation of Proangiogenic ERK and STAT3 Signaling Pathways

**FIGURE 5:**
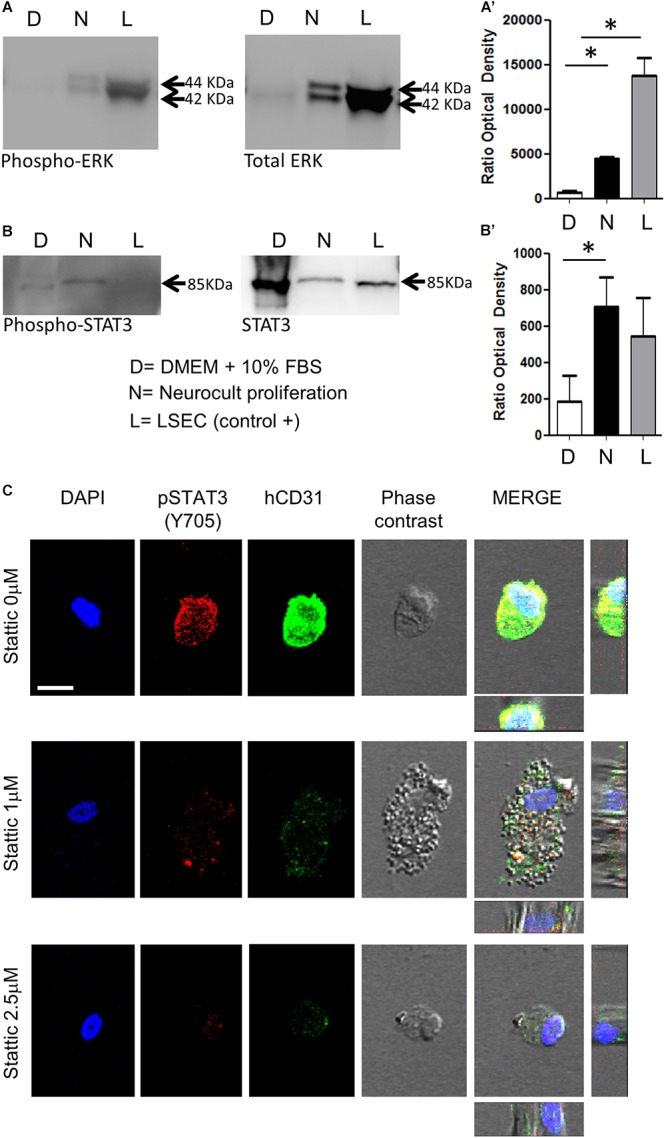
DPSCs grown in Neurocult Proliferation activate both proangiogenic ERK and STAT3 signaling. Western blot membranes of 200,000 cells for each condition showing **(A)** phospho-ERK1/2 and total ERK protein levels and **(B)** phospho-STAT3 and total STAT3 for DPSCs grown either with DMEM + 10% FBS (D) or Neurocult proliferation (N) media. As a positive control, human liver sinusoidal endothelial cells (L, LSEC) were used. **(A’)** Quantification of phospho-ERK1/2 western blot in three different samples for each (D, N, L) condition [mean ± SEM; ^∗^*p* = 0.04 (D vs N) and ^∗^*p* = 0.0273 (D vs L); one-tailed Kruskal–Wallis test]. **(B’)** Quantification of phospho-STAT3 [mean ± SEM; ^∗^*p* = 0.05 (D vs N); one-tailed Kruskal–Wallis test (full blotting of the gel is shown in Supplementary Material)]. **(C)** Immunofluorescence and orthogonal projection of phospho-STAT3 and human CD31 label of DPSCs grown with Neurocult proliferation medium during 72 h using different concentrations of the STAT3 inhibitor Stattic. At concentrations above 1 μM, Stattic abolishes the expression of CD31 in DPSCs. Scale bar 10 μm.

Previous reports demonstrated that endothelial cells differentiation requires STAT3 phosphorylation by a VEGFR2 dependent mechanism (Chen et al., 2008). This VEGFR/STAT3 signaling pathway represents a point of convergence for many angiogenic events (Chen et al., 2008). Once demonstrated the increased presence of phospho-ERK and VEGFR2 in Neurocult-grown DPSCs by PCR and immunofluorescence, we assessed the levels of phospho-STAT3 with respect to total STAT3, as well as phospho-ERK to total ERK by western blot (WB). LSECs were used here as a positive control of mature endothelial cells. As expected, free-floating DPSC dentospheres cultured with Neurocult proliferation presented a higher ratio of phospho-ERK/total ERK band intensity with respect to standard DMEM + 10% FBS conditions [*p* = 0.04 (DMEM + 10% FBS vs Neurocult proliferation and *p* = 0.0273 (DMEM + 10% FBS vs LSEC, Figure 5A,A’)]. Interestingly, the same also applied to the phospho-STAT3/total STAT3 levels, whose ratio was increased specifically in DPSCs grown with Neurocult, with respect to the rest of conditions (*p* = 0.05; one-tailed Kruskal–Wallis test, Figure 5B,B’). Mature LSEC displayed a strong activation of phospho-ERK signaling (Figure 5A) but not of phospho-STAT3 signaling (Figure 5B). Thus, both p-ERK and p-STAT3 signaling pathways were concurrently activated exclusively in Neurocult-grown DPSCs.

### Pharmacological STAT3 Inhibition Abolished Endothelial Cell Induction in Neurocult NS-A-Proliferation-Grown DPSCs

A concurrent activation of ERK and STAT3 has been reported to be necessary for the endothelial cell differentiation of mesenchymal stem cells (Almalki and Agrawal, 2017). Given the evidence that both pathways were active in Neurocult-grown dentospheres, our next step was to disrupt phospho-ERK and phospho-STAT3 signaling to assess whether this was also required for the endothelial differentiation of DPSCs. Because it had been reported that the simple inhibition of ERK basal activity was sufficient to trigger apoptosis in cells cultured without serum (Berra et al., 1998), we decided to focus on the blockage of the STAT3 pathway on DPSCs cultured with Neurocult proliferation. To this end, we chose to test the irreversible inhibitor Stattic (Schust et al., 2006) on DPSCs that had been previously seeded over laminin-coated coverslips. DPSCs cultured with Neurocult proliferation were treated with the inhibitor Stattic for 72 h at concentrations of 0 (control), 1, and 2.5 μM, as previously described (Li et al., 2018). Despite an expected reduction in the number of cells (Villalva et al., 2011), none of the cells in culture in the presence of 1 and 2.5 μM Stattic expressed human-CD31 positive staining (Figure 5C and Supplementary Figures S4A–C). In conclusion, DPSCs grown using Neurocult proliferation media increased concurrently the activity of both ERK and STAT3 signaling pathways, and pharmacological blockade of the latter abolished endothelial cell differentiation of DPSCs in these conditions.

### DPSCs Grown in Neurocult NS-A Differentiation Medium Expressed Neuronal and Astroglial Markers but Not Endothelial CD31

Dental pulp stem cell cultures using Neurocult proliferation media showed increased levels of VEGFR2, CD31+ phospho-STAT3 and phospho-ERK. Similarly, a large body of literature suggested that DPSCs were able to differentiate toward neuronal and astroglial lineages in response to the appropriate stimuli (Gronthos and Zannettino, 2008; Chang et al., 2014; Gervois et al., 2015). Commercial Neurocult media can be optimized for either stem cell expansion or adult neural cell differentiation, by adding their respective culture supplements. Thus, the addition of the differentiation kit supplement to Neurocult medium was already known to induce neuronal and astroglial differentiation of murine NSCs (Lanza, 2006; Belkind-Gerson et al., 2013). In order to test the capacity of neural cell derivation out of DPSCs as compared to NSCs (positive control), both NSCs and DPSCs were grown in parallel with Neurocult differentiation media. Both neurospheres and dentospheres were disaggregated and cells were seeded in laminin-coated coverslips (in adherent form) for 7 consecutive days and then processed for immunofluorescence.

**FIGURE 6:**
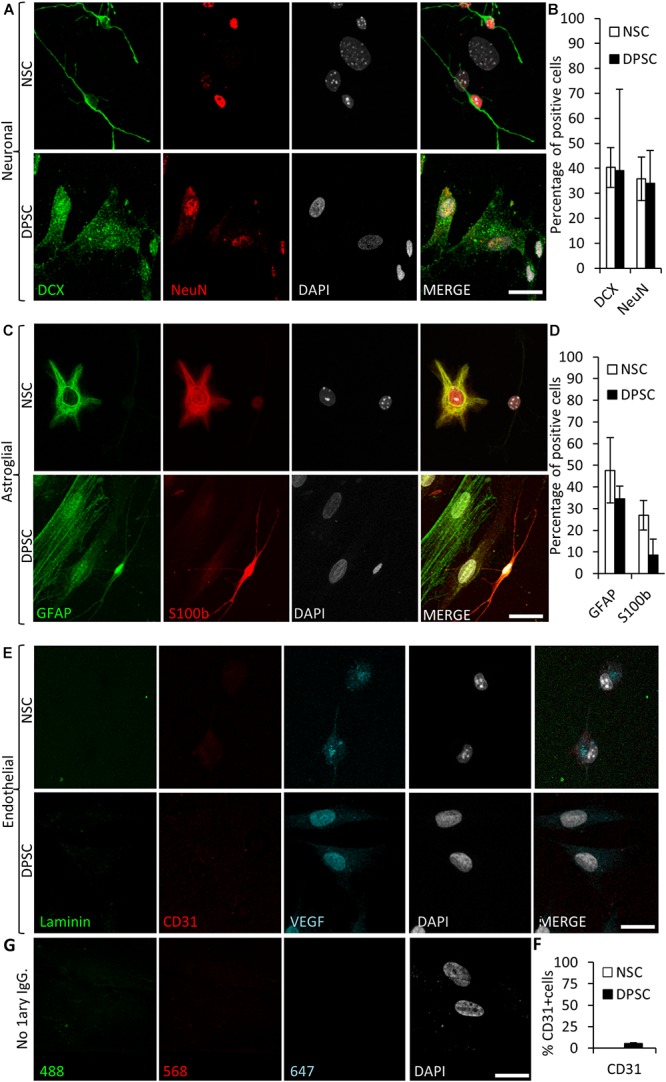
Human DPSCs are able to commit toward differentiation to neuronal-like and glial-like lineages. One week of culture of both human DPSCs and murine (control) NSCs in Neurocult differentiation media is sufficient to induce them express markers for **(A)** neuronal lineage differentiation: doublecortin (DCX) and NeuN staining, for immature and mature neurons, respectively, and **(C)** Astroglial lineage differentiation: glial fibrillary acidic protein (GFAP) and S-100β immunostaining, for immature and mature astrocytes, respectively. **(B,D)** Graphs showing quantifications (mean ± SEM, *n* = 360 cells) of three independent experiments. **(E)** After 1 week of growth in neural differentiation conditions, both NSCs and DPSCs still express VEGF but downregulate CD31. **(F)** Quantification of the proportion of CD31 positive cells (*n* = 571). **(G)** Control with no 1^ary^ antibodies. Scale bar 20 μm.

To assess neuronal lineage differentiation, we used DCX and NeuN markers, both for immature and mature neurons, respectively (Figure 6A). Differentiated NSCs showed a 40 ± 8% of cells positive for DCX and 36 ± 9% for NeuN staining. Differentiated NSC showed a characteristic neuron-like dendritic morphology. On the other hand, when neuronal marker expression was assessed in differentiated DPSCs, 39 ± 33% of cells were positive for DCX, and 34 ± 13% of them showed NeuN staining. However, the morphology of differentiated DPSCs was completely different to differentiated NSCs, with long lamellipodia, and neither presence of dendrites nor any other morphological feature that could make them resemble a neuron. Despite a lack of morphological relationship, we did not find any significant difference between the obtained rates of DCX or NeuN positive cells, (*p* = 0.9701 and *p* = 0.8920, respectively; Student’s *t*-test) depending on the type of source cells employed for differentiation: both DPSCs and NSCs gave rise to neuronal marker-expressing cells in similar proportions (Figure 6B).

In addition, the proportions of cells that differentiated toward astroglial lineage were also assessed. GFAP and S100β were used as markers for immature and mature astrocytes, respectively. It was found that 48 ± 15% of NSCs were positive for GFAP, and 27 ± 7% for S100β staining. On the other hand, 35 ± 6% of human DPSCs showed GFAP staining, and only 9 ± 7% of differentiated DPSCs showed S100β staining. All the cells that showed labeling for S100β also presented bipolar morphology. Again, there were no statistically significant differences in astroglial marker labeling proportions between differentiated DPSCs and NSCs (*p* = 0.0764 Student’s *t*-test, Figure 6C,D).

Finally, we decided to check the generation of endothelial cells in these conditions. Murine NSCs cultured using Neurocult differentiation medium showed cytoplasmic VEGF staining but were negative for the CD31 receptor. In the DPSC counterparts, VEGF expression remained in the nucleus and a dramatic reduction of CD31 labeling, with a scarce 5 ± 1% of CD31-positive cells, were detected (Figure 6E,F). In conclusion, the addition of proliferation supplement to Neurocult medium induced human DPSCs, but not murine NSCs, to express endothelial markers, whereas the addition of differentiation supplement induced both DPSCs and NSCs to generate cells which expressed both immature and mature neuronal and astroglial markers in similar proportions, but with very different morphologies, and a near-absence of expression of CD31.

### DPSCs Grown in Neurocult NS-A Proliferation *in vitro* and Grafted Into the Mouse Brain *in vivo* Migrated and Integrated Into Brain Vasculature

In view of the previous *in vitro* results showing DPSCs being competent to differentiate into endothelial marker-expressing cells using Neurocult NS-A proliferation medium, or switch to neuronal and astroglial marker-expressing cells in Neurocult NS-A differentiation medium, it was important to assess the fate of DPSCs after their *in vivo* grafting into the brain. Because no previous data existed about the generation of brain neovasculature from DPSCs, we decided to carry out *in vivo* intracranial cell grafts using DPSCs grown in Neurocult NS-A proliferation medium. The athymic nude mice model was chosen because it had been previously employed successfully to perform intracranial grafts of human periodontal ligament-derived cells (Bueno et al., 2013). Ten thousand human DPSC cells were grafted as previously described (Pineda et al., 2013) varying the stereotaxic coordinates to place cells intrahippocampally in the dentate gyrus. Cell integration in brain tissue was assessed after 30 days as previously described (Pineda et al., 2013) to better assess the long-term viability of grafted DPSC-derived cells. Human cells were identified using the specific anti-human Nestin antibody as previously described (Jeitany et al., 2015).

**FIGURE 7:**
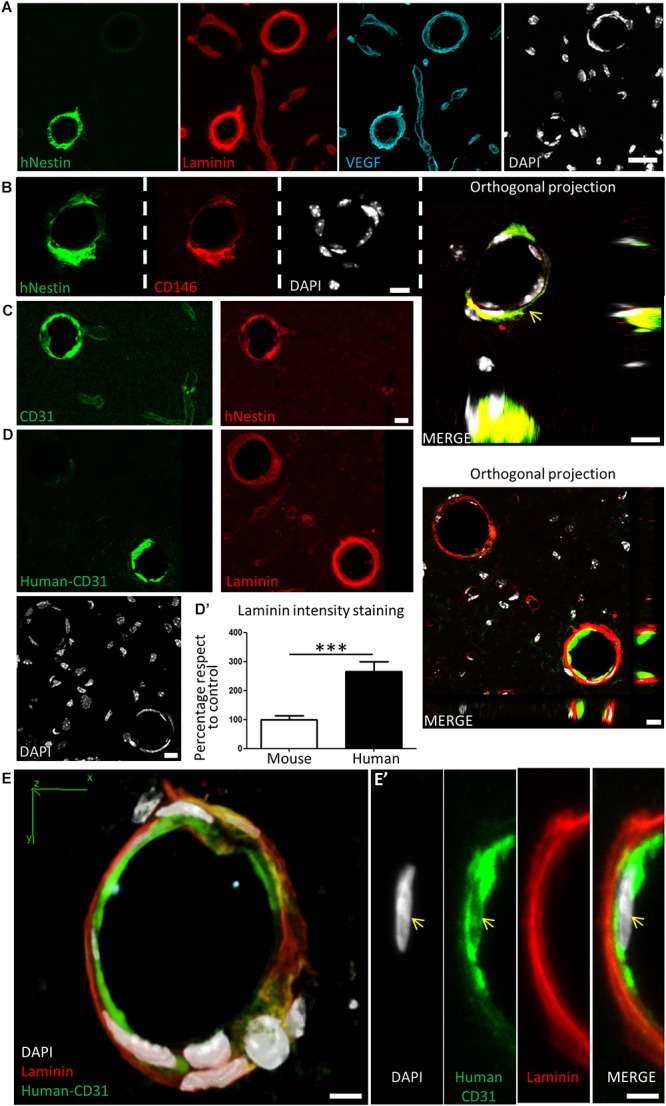
Human DPSCs survive after 1-month post graft into the brain of nude mice, expressing CD31 and VEGF and integrating into the host brain vasculature. **(A)** Detail of human Nestin-positive DPSC-derived cells covering brain vasculature showed an increase in the staining for laminin and VEGF, suggesting *de novo* vasculature formation. **(B)** Triple staining and orthogonal projection of human-Nestin, CD146, and DAPI shows human pericytes wrapping around the endothelial murine (DAPI) cells (arrow). Scale bar 10 μm. **(C)** CD31 staining for both murine and human endothelial cells colocalizes with human Nestin-positive DPSC-derived cells in some medium caliber blood vessels, showing their human origin. **(D)** A specific antibody for human-CD31 cells recognizes human DPSC-derived endothelial cells within some of the blood vessels, which precisely correspond to the ones showing an increased laminin staining. Scale bar of images and orthogonal projection 10 μm. **(D’)** Quantification of laminin staining intensity for murine blood vessels and blood vessels containing human DPSC-derived endothelial cells (mean ± SEM *n* = 30 and 35 blood vessels, respectively, for *n* = 4 grafted animals, ^∗∗∗^*p* = 0.0001, one-tailed Mann–Whitney test). Scale bar 20 μm. **(E)** 3D-reconstruction from a 10-μm thick cryostat slice showing human-CD31 endothelial cells in the inner wall of a blood-vessel decorated with laminin staining. Scale bar 5 μm. **(E’)** Close detail of a human-CD31 positive cell showing the elongated nuclear morphology characteristic of endothelial cells (arrows). Scale bar 5 μm.

Many grafted human cells were located within or very close to blood vessels, which were labeled by laminin and VEGF staining (Figure 7A). Interestingly, some of the small to medium caliber blood vessels (arterioles and venules) appeared to be mostly or exclusively generated by human DPSC-derived cells, showing a very uniform and homogeneous labeling for human proteins in coronal sections. Previous reports demonstrated that Nestin protein is expressed in stem cells, but also in type-2 pericytes and proliferative endothelial cells, but absent in the mature vasculature (Suzuki et al., 2010; Birbrair et al., 2014). Interestingly, recent works using post-mortem human brain tissue reported that human brain capillary-associated pericytes express CD146 (Smyth et al., 2018). We wondered whether our grafted human DPSCs would generate both CD146+ pericyte and CD31+ endothelial cells which could integrate into the murine vasculature. Co-immunolabeling using CD146 (pericyte) or CD31 (endothelia) antibodies was performed together with the human-specific Nestin antibody. By this approach, we detected the presence of both human pericyte cells integrated in the outer layers of the newly generated vasculature (Figure 7B) and also endothelial cells facing the lumen of these human DPSC-derived blood vessels (Figure 7C). To further validate the presence of human endothelial cells derived from DPSC grafts, we proceeded to label serial sections with a human-specific anti-CD31 antibody. Again, we corroborated the presence of human CD31 positive cells integrated into the host brain vasculature of athymic nude mice at 1-month post graft (Figure 7D,E) expressing the endothelial marker CD31 and a characteristic flattened/elongated nuclear morphology, consistent with an endothelial cell identity (Figure 7E,E’).

Close detail allowed us to determine an increased staining of laminin and VEGF in blood vessels containing human cells, with respect to blood vessels only containing the natural murine vasculature (Figure 7A). Remarkably, all the vasculature that contained human cells presented higher amounts of laminin staining. This was quantified by choosing blood vessels of the same caliber, and normalizing the level of intensity of murine vasculature to 100 ± 12%. Thus, we determined that the presence of human DPSC-derived cells within the blood vessel raised the laminin-labeling intensity to 266 ± 33% (*p* < 0.0001 Student’s *t*-test, Figure 7D’). In summary, we demonstrated that human DPSCs, grown with serum-free Neurocult proliferation medium, were able to survive for 1-month and functionally integrate within the brain vasculature of athymic nude mice, expressing markers and morphological vasculature features with characteristics of both pericyte and endothelial cells.

## Discussion

In this work, we reported for the first time that human DPSCs can be grown long-term using serum-devoid Neurocult proliferation media. In these conditions, both murine NSCs and human DPSCs grew as spheres in suspension. Remarkably, the Neurocult NS-A supplemented proliferation medium also increased largely the generation of CD31-positive cells in DPSC cultures. These cells integrated into host nude mice brain vasculature and acquired endothelial cell characteristics, which could be clearly observed after one month post-intracraneal graft. The newly formed vasculature contained both pericyte and endothelial human cells, and included many small to medium size blood vessels which expressed VEGF and had increased levels of laminin staining, suggesting that grafted DPSCs favored a process of angiogenesis and neovascularization. These results open a window to use the DPSCs as a safe source of stem cells to generate endothelial cells which integrate safely and contribute to the generation of complete functional blood vessels into the host brain tissue, thus potentially approaching innovative autologous cellular therapies. To our knowledge, this is the first time in which DPSCs, disaggregated from floating dentospheres grown in a serum-free medium and grafted into the brain of immunosuppressed mice, give rise to CD31-positive endothelial and CD146-positive pericyte cells that can integrate into the host brain vasculature, promoting *de novo* generation of new blood vessels containing both endothelial and mural layers, within brain tissue. Previous works using myocardial infarction model showed that DPSC grafts were able to induce heart angiogenesis (Gandia et al., 2008). Finally, other studies also reported a positive effect of DPSC transplants on the recovery of sensorimotor brain functions after stroke, which was attributed to diverse paracrine mechanisms (Leong et al., 2012). However, in all these cases, DPSCs had been grown in the presence of 10–20% fetal serum.

Previous reports described the capability of sorted DPSCs grown in the presence of 10% fetal serum to differentiate synergistically into osteoblasts and endotheliocytes mantaining their fate when grafted subcutaneously *in vivo* (d’Aquino et al., 2007). However, woven bone chips produced *in vitro* were necessary, contrary to the present results where no bone was generated after the *in vivo* DPSC brain graft. Remarkably, it is known that at a 10–20% concentration, FBS can also stimulate osteoblastic differentiation which in turn promotes the generation of neovasculature (Laino et al., 2006), although bone production is not desirable in the brain. FBS is usually added to the culture medium to support DPSC survival and facilitate cell expansion. This addition imposes at least two big caveats that make serum use very impractical to apply to DPSC transplants to treat brain lesions: (i) the non-desired osteoblastic differentiation of the grafted cells and (ii) the stem cell incorporation and contamination of xenogenic FBS that might cause a dangerous brain inflammatory reaction (Gregory et al., 2006). In the present work, we overcome these limitations using a serum-free culture medium compatible with the intracranial graft of human DPSCs. Neurocult serum-free culture media are routinely used for *in vitro* amplification of NSCs and progenitors and do not cause graft rejection.

Very few works in the literature describe the culture of dental stem cells with no fetal serum in none of the culture phases: expansion and/or differentiation. Hirata et al. (2010) demonstrated for the first time the possibility to maintain stem cells from deciduous or wisdom tooth pulp cells without the need of serum, but without further characterization. Also, Bonnamain et al. (2013) were able to culture human pulp stem cells in a serum-free supplemented basal medium, but they reported a 30% of efficiency to generate non-adherent spheroid-like morphologies (Morsczeck et al., 2010). By a protocol using serum-free Neurocult medium supplemented with proliferation kit and with EGF/FGF2, heparin and B27 without vitamin A, we achieved a near 100% success in dentosphere generation in human DPSC cultures. Previous literature about DPSC intracranial grafts had mainly focused on searching for neuronal fates of the transplanted cells (Arthur et al., 2008; Chang et al., 2014). Our work is the first to report that non-engineered or non-genetically modified human DPSCs can differentiate and integrate into mouse intracerebral vasculature, promoting neovasculogenesis. This approximation for cell expansion without the use of serum is an important aspect for future translational therapies, excluding the potential variability and risk of contamination by xenogenic FBS or genetical manipulation. These results are of major interest because no report exists to date which is focused on obtaining endothelial cells and fully blood vessels starting from DPSCs and using a serum-free protocol. Previous works were focused on the neural differentiation of DPSCs in serum-free media (Widera et al., 2007; Hirata et al., 2010; Bonnamain et al., 2013), or the obtention of endothelial vascular cells using 10% FBS (Weiss et al., 1990; d’Aquino et al., 2007) but none of them addressed the issue of endothelial cell generation out of human DPSCs in the total absence of serum. Non-human vascular cells had already been succesfully cultured without the need of serum, although the reported protocol was not optimized for human cells (Weiss et al., 1990; Gorfien et al., 1993).

Nestin expression had been related to *de novo* formation of vasculature (Suzuki et al., 2010). The coexpression of Nestin and VEGF in grafted human DPSC-derived cells constitutes a solid evidence of neoangiogenesis (Ferrara et al., 2003). Altogether, Nestin/VEGF and/or CD31 human positive cells integrated into the host vasculature with increased laminin staining are the hallmarks that led us to advocate for *de novo* angiogenesis (Nguyen et al., 2000; Malinda et al., 2008). We could speculate that grafts of human DPSC cultured with Neurocult proliferation media could favor a rejuvenation-like benefitial effect in brain vasculature, because laminin is one of the main extracellular matrix (ECM) components that progressively decreases up to 50% during the aging process (Gavazzi et al., 1995). Interestingly, there is also an increase of vascular TGF-β during aging, which has been reported to induce quiescence of neurogenic niches (Pineda et al., 2013). Furthermore, laminin has been described to be able to downregulate TGF-β, at least in epithelial cells (Streuli et al., 1993). Thus, the increase of vascular laminin could again constitute a rejuvenation-like stimulus through TGF-β. From a point of view of biological or therapeutic interest, the increase of vascular laminin observed in blood vessels containing DPSC-derived cells could also have beneficial effects in the case of neurodegenerative illnesses such as Alzheimer’s disease. It has been reported that laminin is able to induce depolymerization of amyloid αβ fibrils reducing the toxic effects of amyloid peptides (Bronfman et al., 1996; Morgan and Inestrosa, 2001). In cerebro-vascular accident models such as focal cerebral ischemia, a degradation of the microvascular matrix of collagen, perlecan and laminin has been reported (Fukuda et al., 2004). In addition, variations of laminin levels during CNS injury have been associated to an attempt to revascularize and oxygenate the tissue (Fukuda et al., 2004; Ji and Tsirka, 2012). In other ischemia models such as the middle cerebral artery occlusion (MCAO), the ECM, and secreted factors such as VEGF are involved in the progressive recovery (Fukuda et al., 2004). *Per se*, the use of DPSCs as a source of cells secreting neuroprotective BNDF also brings an enormous potential for vasculogenesis (Alomari et al., 2015) and neuroprotective therapies (Canals et al., 2004; Pineda et al., 2005). Interestingly, we showed that DPSCs cultured with Neurocult proliferation media maintained *BDNF* expression. This could provide an improvement for neurotrophic therapy in cerebrovascular accidents such as stroke (Schäbitz et al., 2007), a condition which carries a strong economic and social burden (Di Carlo, 2009) together with an increased probability of future dementia (Pendlebury et al., 2019). Furthermore, from a perspective of cellular therapy, brain-grafted DPSCs could compensate the reduction in proliferation of vascular endothelial progenitor cells which is observed after acute ischemic cerebrovascular events (Blum et al., 2019). However, all these discussed applications are beyond the scope of this manuscript, but warrant further investigations.

Interestingly, DPSC expansion in Neurocult proliferation culture media increased the proportion of cells expressing the endothelial markers VEGF and CD31, with respect to neural-differentiated or DMEM/FBS grown DPSC cultures. We also attempted to shed light on the involved mechanism(s) for this effect. It has been reported that constitutive STAT3 activity upregulated VEFG expression (Niu et al., 2002). In agreement, our results clearly showed that Neurocult proliferation medium actually activated the VEGFR/STAT3 signaling pathway in DPSCs, which is fundamental for endothelial cell differentiation. Indeed, we observed a concurrent increase of both phospho-STAT3 and phosphor-ERK levels in Neurocult-grown DPSCs, and this dual concurrent signaling has been associated not only to the activation of endothelial cells (Chen et al., 2008), but also to the ability of mesenchymal stem cells to differentiate to endothelial cell phenotypes (Almalki and Agrawal, 2017). Interestingly, selective inhibition of phospho-STAT3 signaling using the chemical compound Stattic was just enough to abolish the endothelial induction effect of Neurocult proliferation medium in DPSCs. This result highlighted the importance of downstream signaling events to control DPSC endothelial differentiation. Stattic was already known to abrogate both cancer stem cells proliferation (Villalva et al., 2011) and tumor angiogenesis (Niu et al., 2002). Meanwhile, phospho-ERK had been long regarded to play a role in the differentiation of endothelial cells into angiogenic vessels (Xu et al., 2008).

Other dental stem cell types such as SHEDs were also reported to be able to differentiate to neural and astroglial lineages (Miura et al., 2003) and endothelium (Sakai et al., 2010; Bento et al., 2013). Interestingly, the expression of VEGF by SHEDs was required to induce cellular expression of endothelial differentiation markers such as vascular endothelial growth factor receptor-2 (VEGFR2), CD31 (PECAM-1), and VE-Cadherin (Sakai et al., 2010). Similar results have been published with stem cells from the apical papilla (SCAPs) or DPSC previously expanded with α-MEM (d’Aquino et al., 2007; Hilkens et al., 2017). However, the aforementioned studies relied on the presence of 10% FBS in the cell culture medium, and/or the use of hydroxyapatite/tricalcium phosphate 3D scaffolds to show the successful formation of vascularized tissue (Sakai et al., 2010). Our results demonstrate that a serum-free medium designed to culture NSCs is also permissive to grow endothelial cells derived from human DPSC cultures. In our comparative study, we found that murine NSCs in Neurocult proliferation medium presented high levels of phospho-ERK signaling but low levels of phospho-STAT3 signaling, as opposed to human DPSCs grown in the same conditions, which activated both pathways simultaneously. Interestingly, murine NSC were neither able to differentiate toward endothelial cells in the same medium, nor to give rise to functional blood vessels after their graft *in vivo*. Some works have reported the need of estradiol for the endothelial differentiation of neural stem/progenitor cells (Sekiguchi et al., 2013), which might somehow affect these signaling pahways.

## Conclusion

In the present work, we show that DPSCs cultured using Neurocult^TM^ NS-A proliferation supplemented medium increase CD31 and VEGFR2 expression and a concurrent activation of proangiogenic ERK and STAT3 signaling pathways. Pharmacological inhibition of STAT3 is sufficient to impair DPSC differentiation to endothelial cell fate. *In vivo* approach by intrahippocampal graft of DPSCs previously cultured using Neurocult proliferation medium demonstrated that human DPSC-derived endothelial cells migrated and integrated into the host brain vasculature, contrary to murine NSCs. Our results highlight the potential of non-genetically modified human DPSCs grown in serum-free media for cell therapy in future therapeutical approaches for brain vasculopaties. This advance is of utmost importance because the applications of DPSCs in cell therapy have been so far largely limited by the use of fetal serum in conventional DPSC cultures.

## Data Availability

All datasets generated for this study are included in the manuscript and/or the Supplementary Files.

## Author Contributions

JP, FU, JE, and GI contributed to conception and design and financial support. JP, GI, OP-A, FU, and JL contributed to provision of study materials and manuscript writing. JL, OP-A, and JP contributed to collection and/or assembly of data. JL, GI, and JP contributed to data analysis and interpretation. JL, OP-A, JE, FU, GI, and JP contributed to manuscript discussion and final approval of the manuscript.

## Conflict of Interest Statement

The authors declare that the research was conducted in the absence of any commercial or financial relationships that could be construed as a potential conflict of interest.
