# Supplementary material for: Human Dental Pulp Stem Cells Grown in Neurogenic Media Differentiate Into Endothelial Cells and Promote Neovasculogenesis in the Mouse Brain
Source: Front Physiol. 2019 Mar 28;10:347. doi: 10.3389/fphys.2019.00347 (PMC6447688; doi:10.3389/fphys.2019.00347)
Supplement: Supplementary file 1 [file Table_1.DOCX]

Supplementary Material

# Supplementary Figures and Tables

**Supplementary Table 1:** List of primers used in this study.

| **Primers** | | **Sequence 5´-3´** | **Annealing (Cº)** | **Amplicon (bp)** |
| --- | --- | --- | --- | --- |
| β-actin | Upstream | GTTGTCGACGACGAGCG | 58.5 | 93 |
|  | Downstream | GCACAGAGCCTCGCCTT | 59.7 |  |
| Gapdh | Upstream | CTTTTGCGTCGCCAG | 60.3 | 131 |
|  | Downstream | TTGATGGCAACAATATCCAC | 60.8 |  |
| CD31  (PECAM-1) | Upstream | AGATACTCTAGAACGGAAGG | 53.01 | 120 |
|  | Downstream | CAGAGGTCTTGAAATACAGG | 53.03 |  |
| VEGFR2 | Upstream | GTACATAGTTGTCGTTGTAGG | 53.84 | 132 |
|  | Downstream | TCAATCCCCACATTTAGTTC | 52.84 |  |
| VegfA | Upstream | GACCAAAGAAAGATAGAGCAAG | 54.84 | 105 |
|  | Downstream | ATACGCTCCAGGACTTATAC | 53.77 |  |
| Bdnf | Upstream | TTACAAAGCTGCTAAAGTGG | 53.82 | 82 |
|  | Downstream | GAACTGAGATTAGATGGCTTC | 53.82 |  |

CD31 (PECAM-1) = Cluster of differentiation 31 (Platelet endothelial cell adhesion molecule)

VegfA = Vascular endothelial growth factor A

VEGFR2 = Vascular endothelial growth factor receptor 2

Bdnf = Brain-derived neurotrophic factor


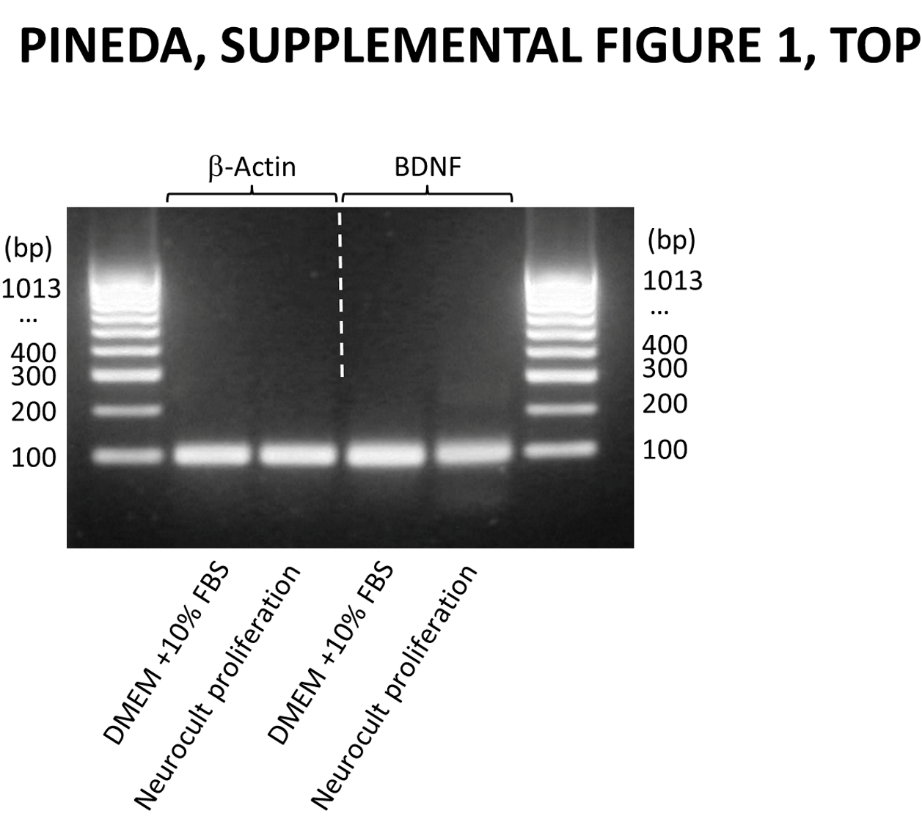


**Supplementary Figure 1:** **DPSCs grown using Neurocult Proliferation media maintain BDNF expression.** Standard RT-PCR shows the expression of the protective neurotrophin BDNF for DPSCs cultured either using DMEM + 10% FBS or Neurocult proliferation media. β-actin is used as loading control. Ladder range is from 100 to 1013 bp.


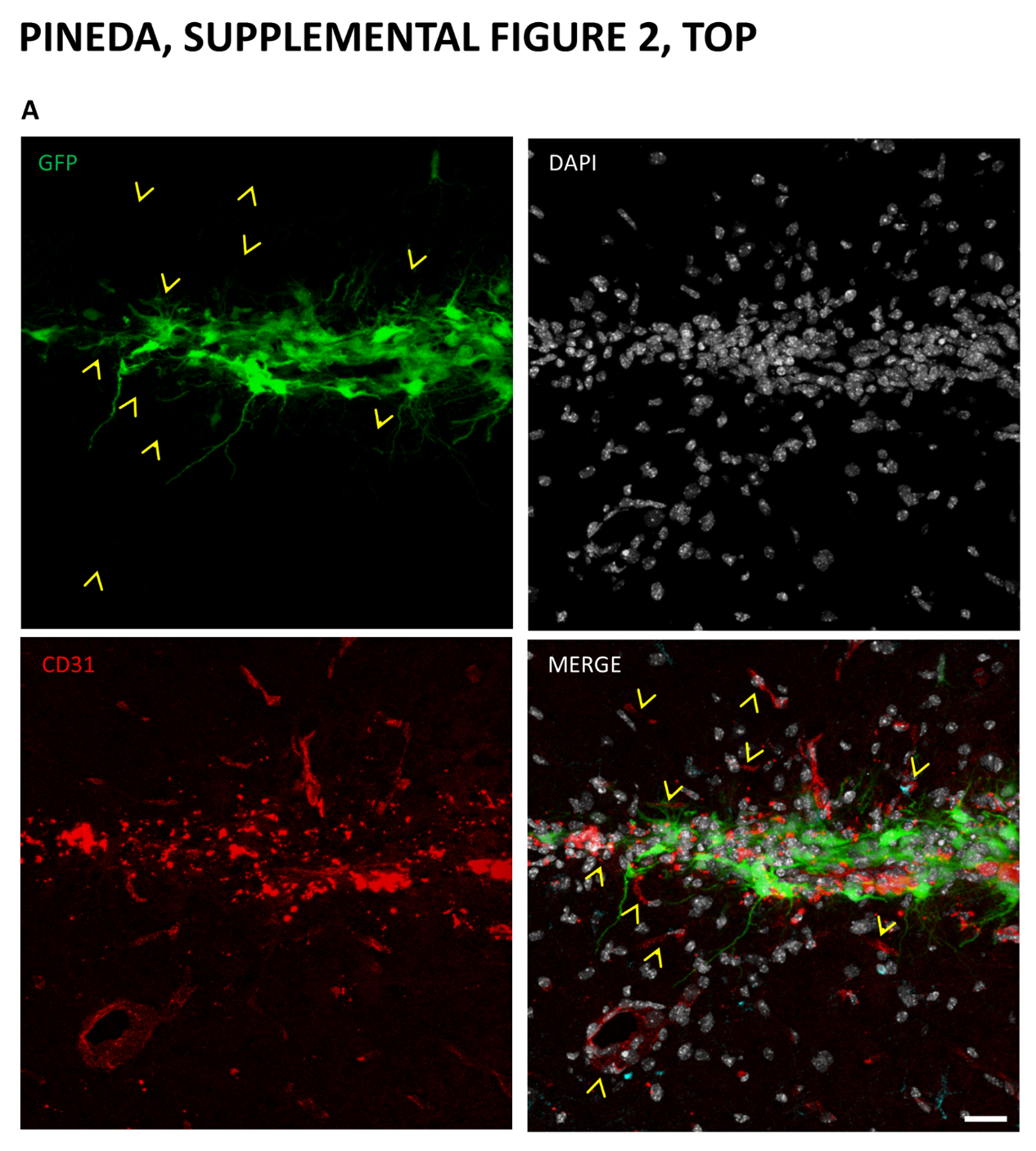


**Supplementary Figure 2: Grafts of consanguineous murine NSCs do not show *in vivo* CD31 staining and cells do not integrate into brain vasculature.**

NSCs isolated from hippocampi of Nestin-GFP positive mice were cultured and expanded using Neurocult proliferation media and ten thousand cells were grafted into the brain of consanguine Nestin-GFP negative littermates. GFP staining allowed a specific labeling of grafted cells. Immunostaining against CD31 was used to label brain vasculature and capillary networks (see arrowheads) but no GFP positive cells colocalized or integrated within host brain vasculature. Scale bar 20 μm.


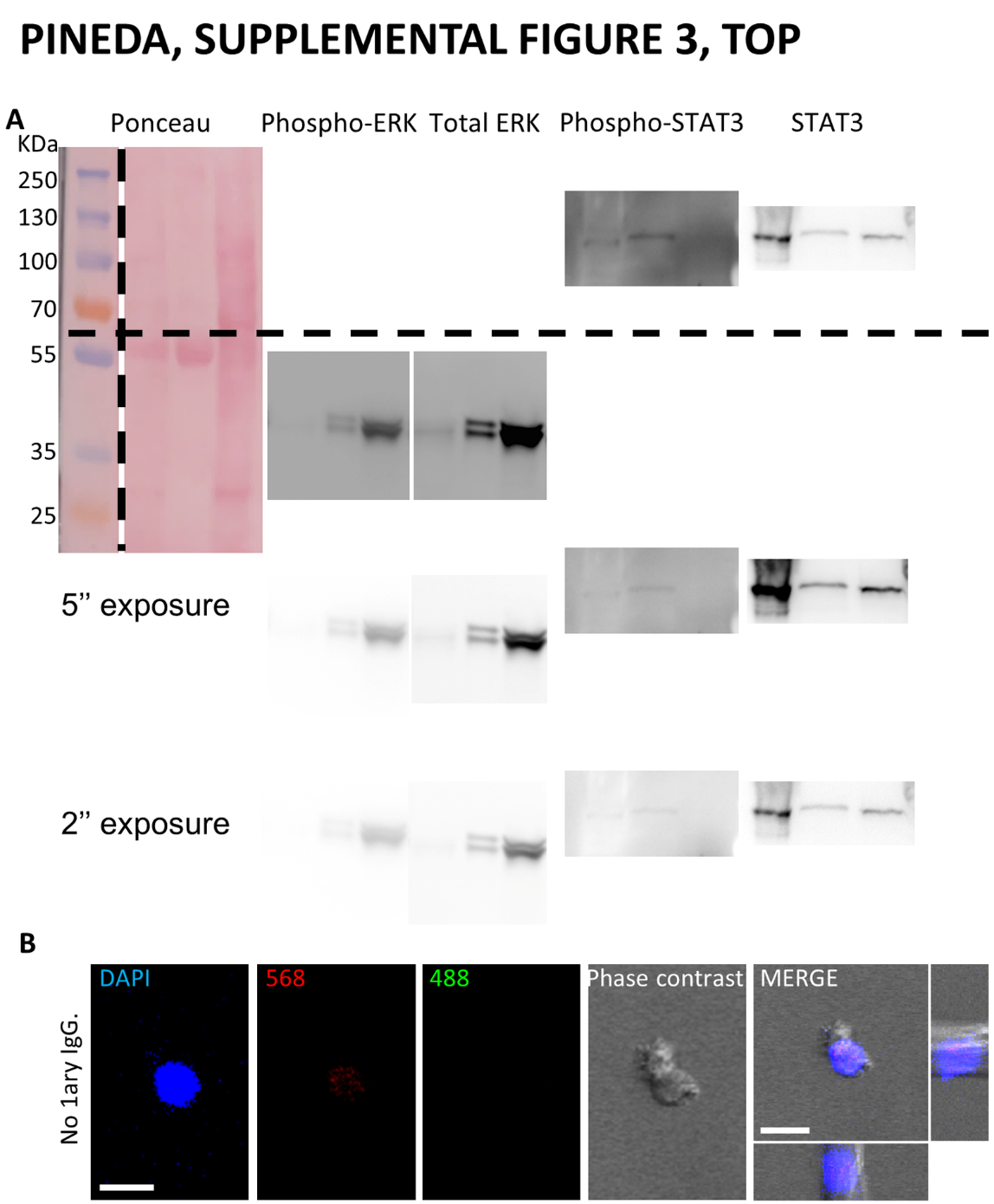


**Supplementary Figure 3: A)** Ponceau staining and blotting of the membrane with Stat3, phospho-ERK and total ERK antibodies corresponding to Figure 4 panel E. Multiple exposures of the blot for the different antibodies are shown and cropped regions of the same membrane are separated with dividing lines. **B)** Negative control without primary antibody for DPSCs grown with DMEM 10% FBS media. Scale bar 75 μm.


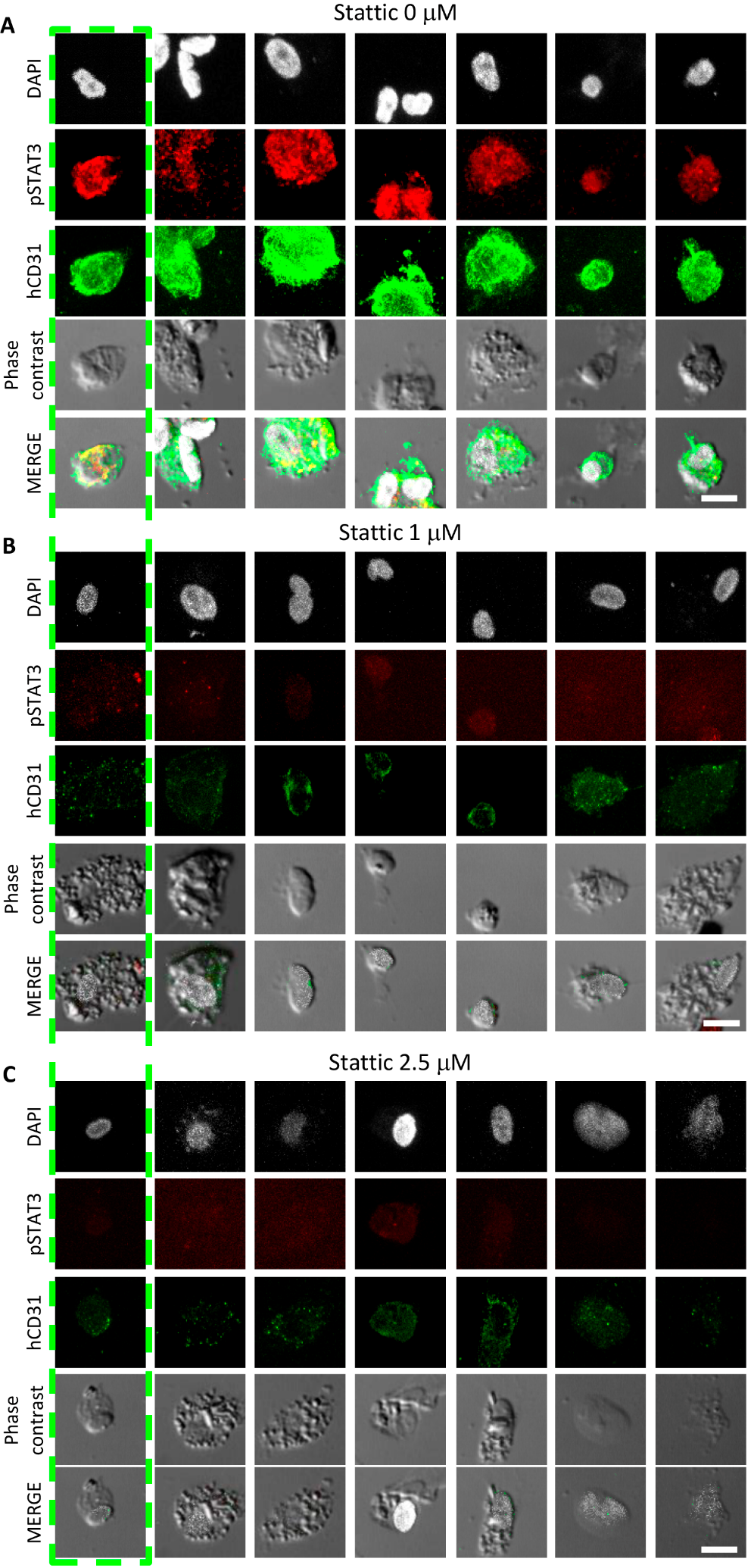
**Supplementary Figure 4:** Additional images of DPSCs grown with Neurocult proliferation media during 72h using different concentrations of the STAT3 inhibitor Stattic. Immunofluorescence against phospho-STAT3 (pSTAT3) and human CD31 (hCD31) label of **(A)** 0μM, **(B)** 1μM and **(C)** 2.5μM of Stattic inhibitor shows that at concentrations above 1µM, Stattic abolishes the expression of CD31 in DPSCs. Please, note that cells of first column (green dotted square) are represented with an orthogonal projection in the Figure 5C. Scale bar 5 μm.
